# Evidence for facultative protocarnivory in *Capsella bursa-pastoris* seeds

**DOI:** 10.1038/s41598-018-28564-x

**Published:** 2018-07-04

**Authors:** Hattie R. Roberts, John M. Warren, Jim Provan

**Affiliations:** 1 Institute of Biological, Environmental and Rural Sciences, Aberystwyth University, Aberystwyth, SY23 3DA UK; 2 Papua New Guinea University of Natural Resources and the Environment, Port Moresby, Papua New Guinea

## Abstract

Many plants derive nutrients by attracting, ensnaring and killing invertebrates, a process that is described as “protocarnivory”. This has been observed in seeds of the weed *Capsella bursa-pastoris*, but it is unclear as to whether it confers any material benefit in terms of germination, establishment and development. In the present study, seeds were germinated in zero, low, medium and high nutrient soils in both the presence and absence of nematodes (*Steinernema feltiae*). Nematodes were attracted to the seeds, with many dying within three days. Germination rates and seedling fresh masses were higher at all nutrient levels, and seedling fresh lengths were higher in all but the zero nutrient treatment, in the presence of nematodes. After transplantation, young plant fresh root lengths and dried leaf and root masses were generally higher in plants that had been germinated in the presence of nematodes across all nutrient levels, with the majority of significant differences being observed in the low-nutrient treatment. Our findings suggest that protocarnivory may play a role in the germination, establishment and early development of *C*. *bursa-pastoris*, and that this process may be facultative, since differences between nematode and non-nematode treatments were generally more pronounced in soils with low nutrient levels.

## Introduction

Since the Gothic era, scientists, explorers and horticulturalists have formed a morbid fascination with carnivorous plants. Darwin’s 1875 book *Insectivorous Plants* enticed botanists, wherein carnivorous mechanisms were explored, and plants as close to home as *Primula sinensis* and *Erica tetralix* were theorized as potentially carnivorous^1,2^. Since Darwin’s findings, Biology’s grisly curiosity has increased, with definitions of botanical carnivory proposed including varying degrees of detail^3,4^. However, there continues to be no clear definition of a carnivorous plant^2^. Simons^3^ suggested that plants must fulfil five criteria: animals must be attracted, trapped and killed, following which enzymes must be secreted, and the plant must absorb products of digestion and derive a material benefit from doing so. Givinish^4^ later proposed a simpler definition of botanical carnivory, namely that plants must attract, ensnare and kill their prey. The lack of solid definitions may also be due to the great diversity in carnivorous morphologies in the plant world; botanical carnivory has been documented to have evolved nine times in twenty eudicot genera^5^. Comparing the evolved mechanisms, from passive pitfalls (*Nepenthes spp*.) to active snap traps (*Dionaea muscipula*), and deeming them “more or less” carnivorous, is impossible. Therefore, it has been suggested that many plants are on a spectrum of carnivory, rather than being definitively carnivorous or non-carnivorous^3^. The term “protocarnivorous” has become increasingly used to describe plants that may not completely fulfil the requirements of “true” carnivory, but use elements of carnivory to aid their establishment, particularly in sub-optimal conditions^5^. Some plants that have recently been proposed as protocarnivorous include *Geranium vissocusum* and *Dipsacus fullonum*^6,7^. However, as with all plant carnivory, it has been argued that there is a cost-benefit model, wherein carnivory tends to evolve in the case that the advantages of carnivory surpass the cost to the plant^4,8^.

There is some evidence to suggest that several species of Brassicaceae, including the model weed *Capsella bursa-pastoris* (Shepherd’s purse), have seeds that possess the attributes that attract, ensnare (i.e. passively capture) and eventually kill small invertebrates^9^. Over the past half century, experiments involving mosquito larvae, nematodes, protozoa, and bacteria have shown positive chemotaxis towards myxospermous *C*. *bursa-pastoris* seeds, and mortality rates of above 90% have been observed upon exposure to damp seeds, particularly in *C*. *bursa-pastoris* and *Descurainia sophia*^9–11^. Mortality in two genera of mosquito larvae (*Culex spp*., and *Aedes spp*., respectively) have been attributed to mouth bristles becoming stuck in the sticky mucilage, causing death due to drowning and exhaustion^9,11^ and, consequently, Barber speculated that *C*. *bursa-pastoris* seeds were arguably carnivorous^10^. However, the seeds are not documented to have an inherent requirement for invertebrates to be present to germinate. Moreover, there is a significant lack of evidence to suggest that *C*. *bursa-pastoris* seeds fulfil either criteria for carnivory described by Simons or Givnish *et al*.^3,4^, and thus “protocarnivorous” may be a more suitable term to describe *C*. *bursa-pastoris*^2^. Whether this mechanism truly acts to the plant’s advantage, i.e. whether *C*. *bursa-pastoris* gains a material benefit, has not been determined. Also unknown is how significantly the protocarnivorous traits help the seed to establish or develop, in terms of germination rate, length or biomass.

The aim of the present study was to determine whether the protocarnivorous traits of *C*. *bursa-pastoris* seeds help them to establish, particularly in low-nutrient soils, since it has been suggested previously that the mechanism of attraction, capture and death of small invertebrates may be advantageous to *C*. *bursa-pastoris* in such conditions^2^. It was hypothesised that (a) germination rate and seedling mass and length of *C*. *bursa-pastoris* would significantly increase in the presence of soil invertebrates; (b) seedlings established in the presence of nematodes would exhibit significantly greater development of leaves and roots compared to those that were not exposed to nematodes, and (c) these increases would be most pronounced in seedlings germinated in low nutrient soils.

## Results

### Nematode attraction to seeds

**Figure 1:**
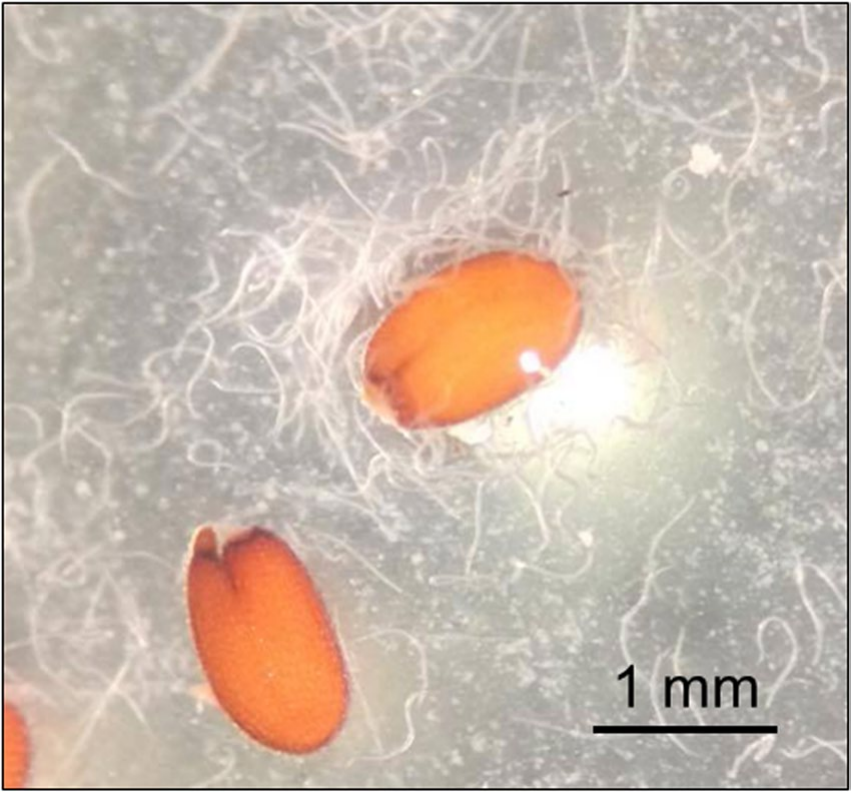
Magnification of *Capsella bursa-pastoris* seeds showing attracted *Steinernema feltiae* nematodes.

Upon first contact with the seeds, the nematodes were not immediately attracted. However, within an hour, many nematodes visibly coated the surfaces of some of the seeds (Fig. 1). Twenty-four hours after initial exposure, it was observed that attraction increased to the mucilage. Three days after initial exposure, many nematodes had died.

### Germination rates and seedling masses and lengths

**Figure 2:**
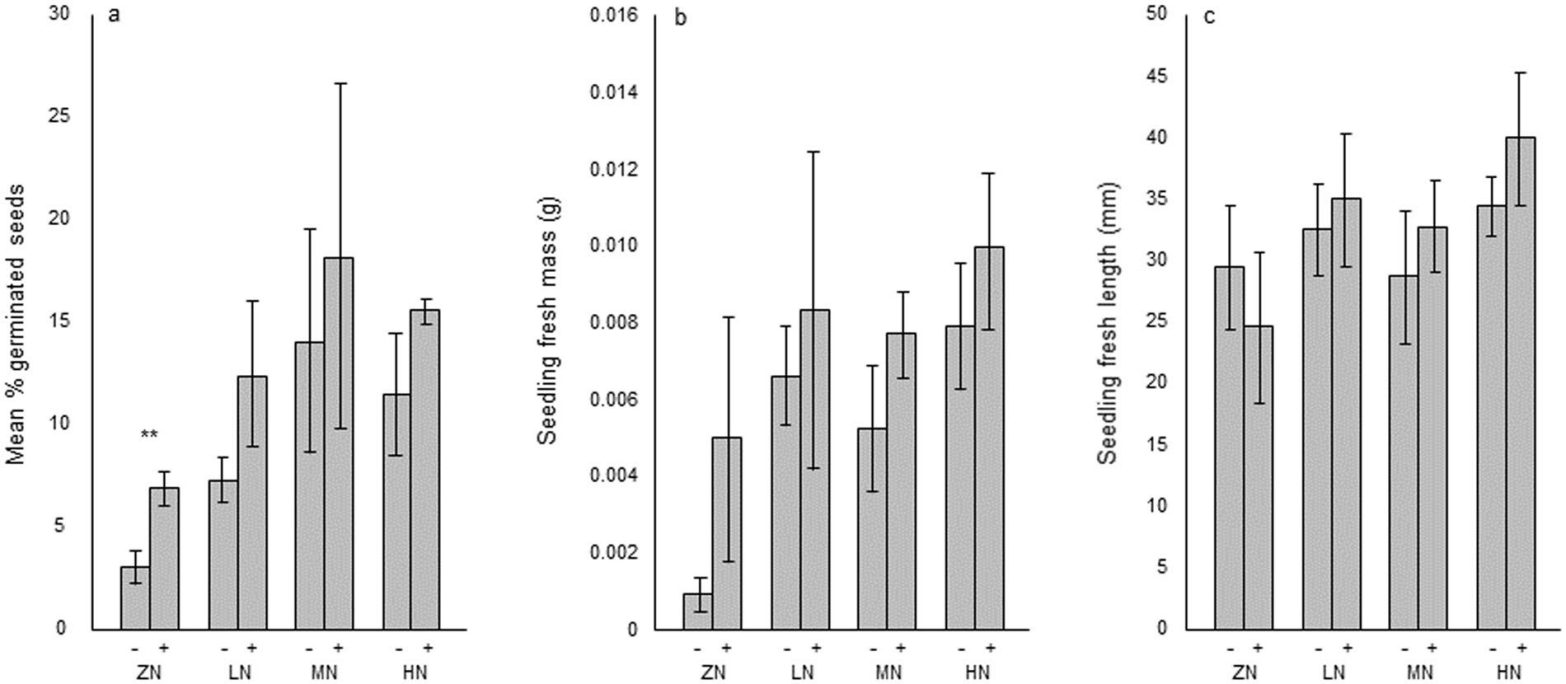
Graphs showing (**a**) Mean % germinated seeds, (**b**) Seedling fresh mass at ten days, and (**c**) Seedling fresh length at ten days at zero (ZN), low (LN), medium (MN) and high (HN) nutrient levels, both in the absence (−) and presence (+) of nematodes. See Methods for details of nutrients.

For brevity and clarity, we have only presented *P* values in the following sections where comparisons were significant. Germination rates at all nutrient levels were higher in the presence of nematodes than in their absence, and ranged from 3.0% ± 0.82 (zero nutrient/nematodes absent) to 17.7% ± 8.18 (medium nutrient/nematodes present; Fig. 2a). The pair of zero-nutrient treatments exhibited significantly different germination rates (3.0% ± 0.82 nematodes absent vs. 7.0% ± 0.82 nematodes present; *P* = 0.008). Seedling fresh masses for all nutrient levels were higher in the presence of nematodes than in their absence, and ranged from 0.0009 g ± 0.0004 (zero nutrient/nematodes absent) to 0.0100 g ± 0.0020 (high nutrient/nematodes present; Fig. 2b). Seedling fresh lengths were higher in the presence of nematodes than in their absence at all nutrient levels with the exception of the zero nutrient experiment, and ranged from 23.9 mm ± 6.31 (zero nutrient/nematodes present) to 40.4 mm ± 5.57 (high nutrient/nematodes present; Fig. 2c).

### Young plant fresh root lengths and dried leaf and root masses

**Figure 3:**
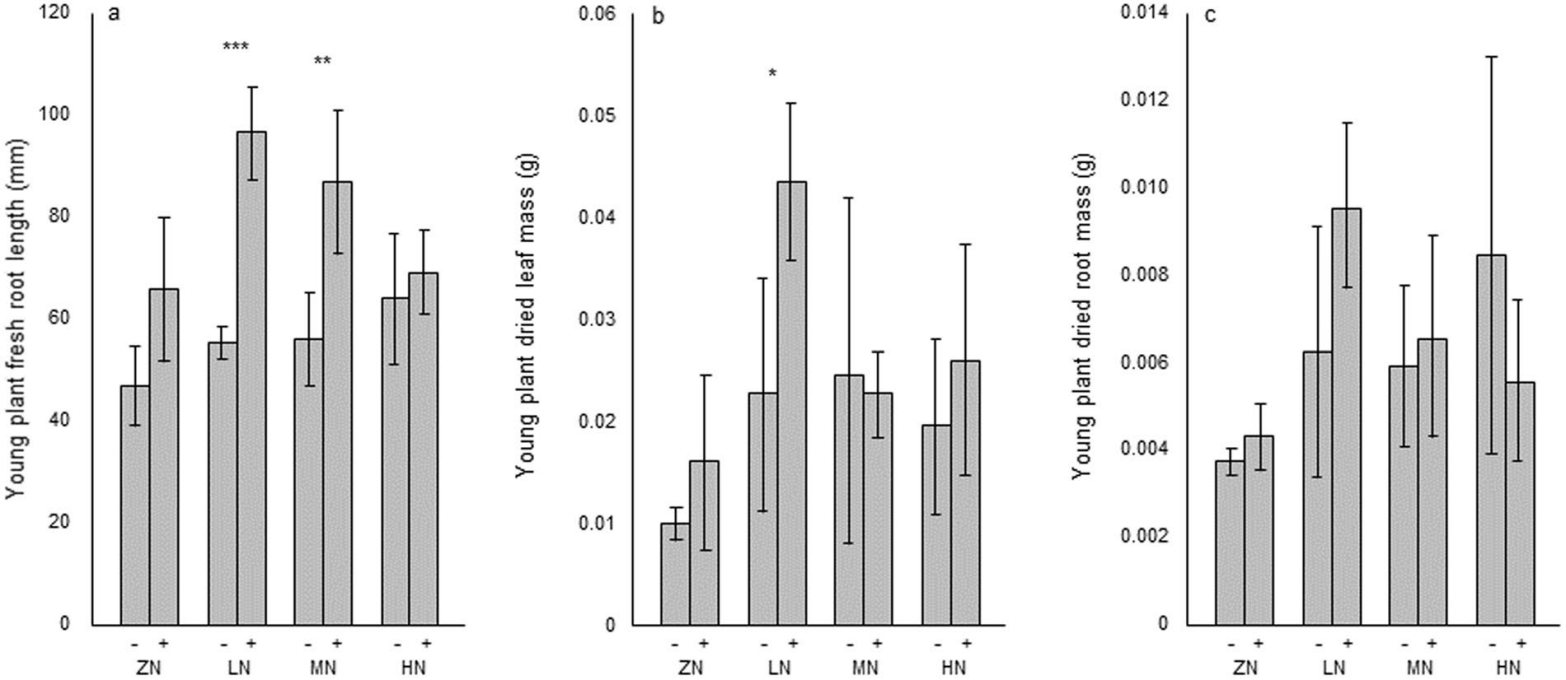
Graphs showing (**a**) Young plant fresh root length, (**b**) Young plant dried leaf mass, and (**c**) Young plant dried root mass after germination at zero (ZN), low (LN), medium (MN) and high (HN) nutrient levels, both in the absence (−) and presence (+) of nematodes. See Methods for details of nutrient levels.

Fresh root lengths were longer in young plants germinated in the presence of nematodes than in those germinated in the absence of nematodes for all nutrient levels (Fig. 3a). Lengths ranged from 46.3 mm ± 7.2 (zero nutrient/nematodes absent during germination) to 97.0 mm ± 10.4 (low nutrient/nematodes present during germination). Values for plants grown in low- and medium-nutrient conditions were significantly higher for plants germinated in the presence of nematodes (97.0 mm ± 10.4 and 87.8 mm ± 15.7 respectively) than for those germinated in the absence of nematodes (55.3 mm ± 5.6, *P* < 0.001 and 57.1 mm ± 12.1, *P = *0.010 respectively).

Dried leaf masses were higher in young plants germinated in the presence of nematodes than in those germinated in the absence of nematodes for all but the medium nutrient levels (Fig. 3b). Masses ranged from 0.011 g ± 0.002 (zero nutrient/nematodes absent during germination) to 0.043 g ± 0.010 (low nutrient/nematodes present during germination). The latter value was significantly higher than that for plants germinated in the absence of nematodes and grown at the same nutrient level (0.023 g ± 0.010, *P* = 0.028).

Dried root masses were higher in young plants germinated in the presence of nematodes than in those germinated in the absence of nematodes at zero, low and medium nutrient levels (Fig. 3c). Values ranged from 0.0035 g ± 0.0003 (zero nutrient/nematodes absent during germination) to 0.0097 g ± 0.0020 (low nutrient/nematodes present during germination).

## Discussion

The findings of the present study suggest that protocarnivory may play a role in the germination, establishment and early development of *Capsella bursa-pastoris*. Nematodes exhibited attraction to the seeds, and germination rates and seedling mass and length were higher in the presence of nematodes. Development was generally more pronounced in young plants that had been germinated in the presence of nematodes than in their absence. Protocarnivory in *C*. *bursa-patoris* may be viewed as faculatative, since differences between treatments were generally greater in soils with lower nutrient levels.

Whilst positive chemotaxis exhibited by *S*. *feltiae* towards most *C*. *bursa-pastoris* seeds was clearly observed, the mechanisms underlying nematode olfaction are complex and mostly undetermined. There is evidence of olfactory adaption to chemoattractants occurring in some species^12^, and nematodes have been observed to be attracted to plant volatile compounds in the Fabaceae family, mediating colonization of symbiotic bacteria into root nodules^13^. However, it is not known whether secondary compounds play any role in the protocarnivorous mechanism of *C*. *bursa-pastoris*. It was proposed by Barber *et al*. that the attraction and attachment mechanism of *C*. *bursa-pastoris* may be multifunctional^14^, but whilst it is possible that polysaccharides function as the attachment mechanism, it is less likely that their hydrolysed derivatives act as a chemoattractant. Previous work has demonstrated that *Culex pipiens quinquefasciatus* (mosquito) larvae are attracted to *C*. *bursa-pastoris*^14,15^, and it was proposed that whilst the mucilage of the seeds acted as a source of nutrients for the larvae, positive chemotaxis was towards certain nucleotides found in the seed mucilage^15^, since RNA and its hydrolysed derivatives have been observed to act as chemoattractants^16,17^.

Other species of the Brassicacaea family have also been documented to attract and ensnare mosquito larvae upon imbibition^18^. Reeves & Garcia recorded that *Descurainia sophia*, *D*. *pinnata*, *Lepidium flavum* and *Hirschfeldia incana* (syn. *Brassica geniculata)* also exhibited potentially protocarnivorous properties in attracting and retaining *Culex tarsalis* and *C*. *peus*^9^. *D*. *sophia* and *D*. *pinnata* were documented to be more efficient that *C*. *bursa-pastoris* at attracting and ensnaring mosquito larvae, with 95% and 94% of larvae being attached to *Descurainia* seeds, compared to 86% of *C*. *bursa-pastoris*^9^. Eshita et al. also observed that *Brassica campestris rapifera* cv. Yorii-kabu, *Brassica campestris oleifera* cv. Natane, *Cardamine scutata*, as well as *C*. *bursa-pastoris* attracted and captured *Aedes albopictus* in their mucilage^14^. However, only *C*. *bursa-pastoris* was considered protocarnivorous, having been the only species used in later experiments^10^.

The key to the proposed protocarnivorous mechanism may lie in the cellulose content of mucilage of the *C*. *bursa-pastoris*. There is a relatively high cellulose composition (40.9%) recorded in the mucilage of the 1 mm seeds, compared to other small mucilaginous seeds in the same family, and acidic polysaccharides such as rhamnose have also been recorded in the seed mucilage^19^. It has been suggested that there is a correlation between presence of cellulose in the mucilage, and the ability for a seed to trap larvae^10,19^. The presence of cellulose may cause the seed mucilage to become ‘sticky’ upon imbibition, providing a matrix for the acidic branched polysaccharides to bind to^15,19^.

The proposed toxic compounds released upon imbibition of seeds of *C*. *bursa-pastoris* have not been specifically identified. Nevertheless, the Brassicaceae family are known to produce an array of secondary metabolites, including glucosinolates, which, when hydrolysed to isothiocyanates, deter generalist pests, but may attract specialist pests, and have been documented as nematocidal^20,21^. Seed cells can possess myrosinase, an enzyme which hydrolyses glucosinolates into toxic isothiocyanates^20^. However, it is not known whether glucosinolate compounds play any role in the protocarnivorous mechanism of *C*. *bursa-pastoris*. Alternatively, it may be that saprotrophic microorganisms that may facilitate the breakdown of the captured invertebrate matter, similarly to *Brocchinia* species using commensal microbes to break down prey matter^5^. Therefore, nutrients may be hydrolysed and reduced, and become available to young *C*. *bursa-pastoris* seedlings, thus increasing likelihood of survival.

Young plants germinated in the presence of nematodes exhibited enhanced development, suggesting that established *C*. *bursa-pastoris* seedlings derived a nutrient benefit from the presence of the nematodes. This was particularly notable in those germinated in the low nutrient soil, where both dried leaf mass and fresh root length (but not dried root mass) were significantly higher in the nematode-treated plants. The observation of biomass allocation in leaves of the plant, supports the theory that protocarnivory during establishment may increase photosynthetic output^17^. The increase in root length could be considered surprising, however, since plants with carnivorous properties have generally been documented to allocate biomass to leaves rather than roots^4^, although partitioning of prey-derived nitrogen into above and below-ground biomass has been observed in carnivorous plants in low nutrient habitats^22^. Thus, changes in resource allocation may be facultative, with different partitioning of prey-derived nutrients dependent on the nutrient content of the soil during establishment. Furthermore, the patterns of nutrient allocation suggested for “true” carnivorous plants have not been researched in protocarnivorous plants, further implying that a spectrum of carnivory, rather than a purely binary definition (carnivorous vs. non-carnivorous), occurs in nature^3^. Indeed, increased understanding of carnivorous and protocarnivorous plants may mean that terms such as “cryptocarnivory” may become increasingly common, reflecting the fact that botanical carnivory does not have rigid definitions.

## Methods

Seeds were collected from the buffer strip of a managed field of Brassica napus in the valley of Walton, Powys, East Wales (52°13′38.94′′N; 3° 4′0.92′′W) on 24th June and 24th July 2016. Germination tests were carried out in a Conviron 8 growth chamber (Conviron Europe ltd. Cambridgeshire, U.K.). Optimum growing conditions were established at 23 °C, 40 µmol m^−2^ s^−1^, 16 hr light, 8 hr darkness at 10 °C. Although these represent very low light levels, to further minimize any confounding effects of phototaxis in the nematodes, the growth room lacked windows and lights were situated directly overhead. Germination trials lasted ten days. Zero-nutrient control conditions were created by using a base of filter paper to prevent desiccation of the seeds. 10 g of low nutrient (Levington’s M2: pH 5.3–6.0; N-144; P-73; K-239; Peat 0–5 mm; 95%), medium nutrient (John Innes no. 2: nutrient content unavailable), and high nutrient (Levington’s M3: pH 5.3–6.0; N-233; P-104; K-339; Peat 0–5 mm; 100%) compost were used to create corresponding conditions. Petri dishes were kept relatively damp using distilled water in a pressure sprayer (1.25 L Hozelock Spraymist), which prevented the seeds from desiccating during germination. 100 seeds were placed in 90 mm petri dishes with the soils, with or without nematodes (*Steinernema feltiae*; Dragonfli Ltd.). Nematodes were dissolved at a concentration of 5,000 per ml in distilled water, and 3 ml was pipetted onto the soil/filter paper in petri dishes. Petri dishes were checked daily and photographed regularly. Germinated seeds were checked/counted using the naked eye.

Seedlings were subsequently removed from the petri dishes at ten days old and grown on in 3 × 3 pack bedding containing John Innes no. 2 compost to investigate whether their early/more successful establishment affects maximum growth potential. Seedlings were grown for a further 21 days in a glasshouse under a 14 h/10 h light/dark regime, and watered daily using tap water. Up to nine plants were harvested from each treatment group. Roots were washed using a hose with applied pressure and longest roots were measured. Above-ground and below-ground masses were separated with surgical scissors and dried in an oven (Jouan EU3; Innovens^TM^, Thermo Fisher Scientific, U.K.) at 60 °C for approximately 108 hours. Samples were then removed and plant/root biomasses were weighed.

### Data availability statement

The datasets generated during and/or analysed during the current study are available from the corresponding author on reasonable request.
